# Oral intake of royal jelly improves anti-cancer effects and suppresses adverse events of molecular targeted therapy by regulating TNF-α and TGF-β in renal cell carcinoma: A preliminary study based on a randomized double-blind clinical trial

**DOI:** 10.3892/mco.2020.2148

**Published:** 2020-09-25

**Authors:** Yasuyoshi Miyata, Kyohei Araki, Kojiro Ohba, Tomhiro Mastuo, Yuichiro Nakamura, Tsutomu Yuno, Yuta Mukai, Asato Otsubo, Kensuke Mitsunari, Yasushi Mochizuki, Hideki Sakai

Mol Clin Oncol 13:29, 2020; DOI: 10.3892/mco.2020.2099

Following the publication of this article, the authors have realized that, in the Materials and methods section, in the subsection ‘*Protocol*’ on p. 2, the second and the final sentences of the second paragraph contained errors: The text in the second paragraph of this subsection should have been written as follows: ‘RJ was procured from Yamada Agriculture Center Inc (Okayama, Japan). RJ and placebo were prepared as capsules containing **800** mg RJ and starch, respectively. They were similar in taste, smell, size, shape, and color. Capsules were administered orally **three** times per day (after breakfast, lunch, and dinner) for three months.’ [NB. - it was incorrectly stated that the capsules contained 900 mg RJ, and the final sentence incorrectly stated that capsules were administered orally four times a day, including before bedtime.]

The authors regret that this error went unnoticed prior to the publication of the article, and apologize to the readership of the Journal for any inconvenience caused.

